# Operationalization of bi-directional screening for tuberculosis and diabetes in private sector healthcare clinics in Karachi, Pakistan

**DOI:** 10.1186/s12913-019-3975-7

**Published:** 2019-03-06

**Authors:** Mashal S. Basir, Shifa S. Habib, Syed M.A. Zaidi, Saira Khowaja, Hamidah Hussain, Rashida A. Ferrand, Aamir J. Khan

**Affiliations:** 1 Interactive Research & Development, 4th Floor, Woodcraft Building, Plot No. 3 & 3-A, Sector 47, Korangi Creek Road, Karachi, Pakistan; 2 Community Health Solutions, 9th Floor, Al-Tijarah Building, Main Shahrah-e-Faisal, Karachi, Pakistan; 3 Interactive Research & Development, London School of Hygiene and Tropical Medicine, Keppel St, Bloomsbury, London, WC1E 7HT UK

**Keywords:** Bi-directional screening, Tuberculosis, Diabetes, Active-case-finding, Operational constraints

## Abstract

**Background:**

Many countries are facing overlapping epidemics of tuberculosis (TB) and diabetes mellitus (DM). Diabetes increases the overall risk of developing Tuberculosis (TB) and contributes to adverse treatment outcomes. Active screening for both diseases can reduce TB transmission and prevent the development of complications of DM. We investigated bi-directional TB-DM screening in Karachi, Pakistan, a country that ranks fifth among high TB burden countries, and has the seventh highest country burden for DM.

**Methods:**

Between February to November 2014, community-based screeners identified presumptive TB and DM through verbal screening at private health clinics. Individuals with presumptive TB were referred for a chest X-ray and Xpert MTB/RIF. Presumptive DM cases had random blood glucose (RBS) tested. All individuals with bacteriologically positive TB were referred for diabetes testing (RBS). All pre-diabetics and diabetics were referred for a chest X-ray and Xpert MTB/RIF test. The primary outcomes of this study were uptake of TB and DM testing.

**Results:**

A total of 450,385 individuals were screened, of whom 18,109 had presumptive DM and 90,137 had presumptive TB. 14,550 of these individuals were presumptive for both DM and TB. The uptake of DM testing among those with presumptive diabetes was 26.1% while the uptake of TB testing among presumptive TB cases was 5.9%. Despite efforts to promote bi-directional screening of TB and DM, the uptake of TB testing among pre-diabetes and diabetes cases was only 4.7%, while the uptake of DM testing among MTB positive cases was 21.8%.

**Conclusion:**

While a high yield for TB was identified among pre-diabetics and diabetics along with a high yield of DM among individuals diagnosed with TB, there was a low uptake of TB testing amongst presumptive TB patients who were recorded as pre-diabetic or diabetic. Bi-directional screening for TB and DM which includes the integration of TB diagnostics, DM screening and TB-DM treatment within existing health care programs will need to address the operational challenges identified before implementing this as a strategy in public health programs.

## Background

Nearly one third of the world’s population is infected with *Mycobacterium tuberculosis (Mtb)* [1]. A growing body of literature has established the association between tuberculosis (TB) and diabetes mellitus (DM) in various settings [2]. DM increases the overall risk of developing TB and contributes to adverse TB treatment outcomes such as delayed sputum conversion, treatment failure and relapse and death [2]. The global burden of diabetes is on the rise with the total number of people living with DM estimated to reach 438 million by 2030 [3]. The convergence of these two epidemics poses a significant challenge for health systems in low and middle-income countries. Studies in India and China found significant evidence for comorbidity at various health care levels including tertiary care facilities and primary healthcare clinics [4, 5]. These studies led to important policy formulations that have mandated screening for DM among all TB patients and documentation of DM indicators within registries of the TB control program in these countries.

Screening, early-case detection and prompt treatment initiation is of relevance for both diseases. In 2009, the World Health Organization (WHO) and the International Union Against Tuberculosis and Lung Disease (IUATLD) recommended bi-directional TB-DM screening and integrated management for both diseases in high burden countries [6]. However, in low and middle-income countries, health systems are often weak and highly fragmented, including large and often poorly regulated private-sectors, and are not equipped to respond effectively to the dual burden of communicable and non-communicable diseases [7].

Pakistan is currently ranked fifth among high TB burden countries [1]. Nearly half of all cases remain undiagnosed and improving case-detection is an important target for the National TB Control Program (NTP) [1]. Pakistan also faces a rising burden of non-communicable diseases (NCDs), particularly Type II DM. It has the seventh highest country-burden for DM with an estimated prevalence of 6.8% in the adult population [8, 9]. Similar to TB patients, diabetics are frequently under-diagnosed and an estimated 3.5 million adults in Pakistan are thought to be living with undiagnosed DM [9]. In Pakistan, TB control activities are under the purview of the National Tuberculosis Program (NTP), but routine screening for NCDs including DM is not currently recommended. Interventions also need to consider the private-sector that provides healthcare services to nearly three-quarters of all patients in the country [10].

In recent years, novel case-finding approaches for TB in the private-sector have been developed to enhance case-notifications through community-based screening, cash-incentives for referrals and linking case-notifications to the NTP disease surveillance system [11, 12]. Similar public-private models with a focus on integrated care can lower costs and improve health outcomes by shifting diseases-specific interventions to holistic, patient-centric approaches [13]. We investigated the feasibility of a bidirectional TB and DM screening program in the private health sector in Karachi, Pakistan as part of an enhanced case-finding intervention for TB through a social business approach (described below). The initiative builds upon previous interventions carried out in the area that were limited to targeting community-based screening for TB.

## Methods

### Study design and setting

A cross-sectional study of a bi-directional TB-DM screening program was conducted within a larger study investigating enhanced case-finding intervention for TB in the towns of Korangi, Landhi, Orangi, North Karachi and Nazimabad in Karachi, Pakistan, between February to November 2014. Community health workers (CHW) screened patients attending 80 general primary care, health provider clinics for TB and DM. The screening was based on a convenience consecutive sample. These clinics were primarily privately-owned facilities of individual physicians and a small number of physician private group-practices, serving 50 or more patients on average per day.

### Screening and testing procedures

CHWs were trained on TB screening and treatment according to NTP guidelines, and on DM screening according to the International Diabetes Federation (IDF) [14]. CHWs were subsequently assigned to private health providers’ clinics where they verbally screened attendees for presumptive TB and DM. CHWs and health care providers were incentivized for referrals and case-detection. Presumptive TB was defined as cough of any duration, fever, unexplained weight-loss, night-sweats or hemoptysis. Patients with presumptive DM were defined as those aged over 30 years and with a family history of DM.

TB testing was carried out at purpose-built diagnostic centers called *Sehatmand Zindagi* (Healthy Life) located in Korangi and Nazimabad. The centers operate as a social business that aim to provide sustainable TB care in the private-sector. Revenue is generated through direct payment for tests by patients, including chest X-rays (USD 3–5 per test), spirometry (USD 2.5–5 per test) and random blood glucose testing (USD 0.3–0.5 per test). Xpert MTB/RIF testing was provided free-of-cost to all patients following a chest X-ray. The centers served as the site of treatment initiation and case-registration to the Provincial TB Program (PTP). The revenue generated is channeled into subsidizing TB diagnosis and treatment.

**Fig. 1:**
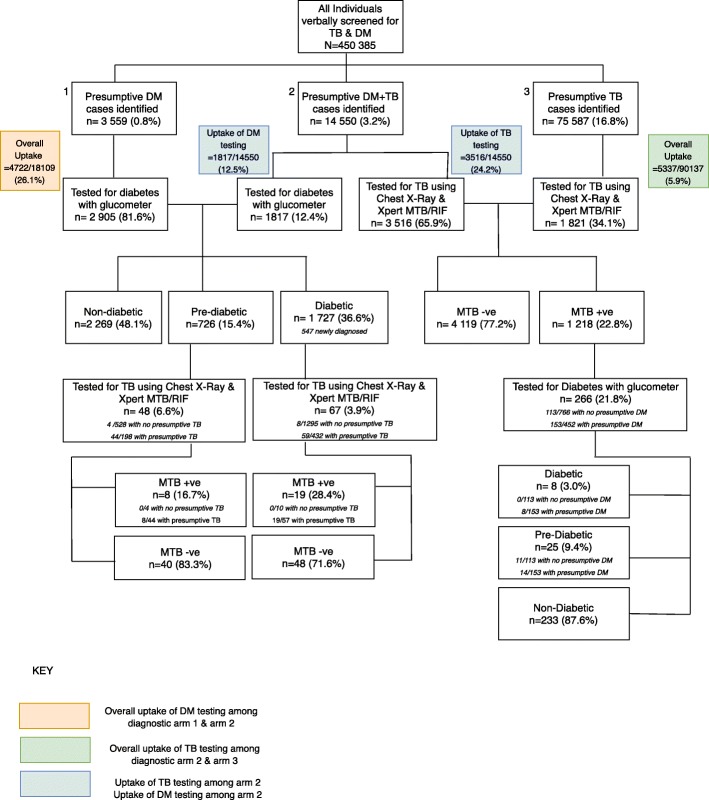
Diagnostic algorithm and results of TB-DM bidirectional screening Feb-Nov, 2014, Karachi, Pakistan

Individuals with presumptive TB were referred by the private health provider to the social business TB centers for a paid chest X-ray, followed by a free Xpert MTB/RIF test. Patients with bacteriologically positive TB were tested for DM through a glucometer-based random blood sugar test (RBS) at the diagnostic centers. Individuals who came to the Centers with a previous diagnosis of TB or DM were re-tested using *Sehatmand Zindagi* Centers’ Chest X-ray, Xpert MTB/RIF and glucometer-based random blood sugar tests to ensure reliable baseline measures for diagnosis rather than depending on external reports. 1180 individuals with known diabetes (Table 1), defined as all those who self-reported to be taking oral hypoglycemic medication, underwent glucometer based random blood sugar testing and then were channeled into the presumptive DM-TB arm (Fig. 1). Baseline data for previously diagnosed and newly diagnosed diabetes cases is indicated in Table 1. Previously diagnosed cases of diabetes were further classified as having controlled or uncontrolled diabetes. For the purpose of this analysis, uncontrolled diabetes was defined as RBS of greater than 200 mg/dl on glucometer testing. Those with presumptive DM were offered a paid (US $2), point-of-care capillary blood-glucose test that was conducted by CHWs at the clinics. Cutoffs recommended by the International Diabetes Federation for resource-limited settings were used to categorize the patients as having DM (≥200 mg/dL (11.1 mmol/L)), Pre-DM (140–199 mg/dL (7.8–11.0 mmol/L)) or no diabetes (< 140 mg/dL (7.8 mmol/L)). Individuals with both presumptive TB and DM, were assigned to another diagnostic arm where the private health provider referred patients for both tests: a point of care glucometer test for DM and Chest X-ray and Xpert MTB/RIF testing at the diagnostic centers.

**Table 1:** Baseline characteristics of patients identified as pre-diabetics and diabetics through community-based screening

Characteristics	Cases diagnosed with Pre-diabetes		Newly diagnosed cases of Diabetes		Previously known cases of Diabetes				Total	
					Uncontrolled		Controlled			
	*n*	%	*n*	%	*n*	%	*n*	%	*n*	%
Total Number of patients	726	100	547	100	901	100	279	100	2453	100
Sex										
Male	334	46	236	43.1	359	39.8	100	35.8	1029	41.9
Female	392	54	311	56.9	542	60.2	179	64.2	1424	58.1
Age										
< 30	74	10.2	29	5.3	28	3.1	28	10	159	6.5
30–39	195	26.9	98	17.9	129	14.3	51	18.3	473	19.2
40–49	221	30.4	176	32.2	284	31.5	58	20.8	739	30.2
50–59	135	18.6	151	27.6	284	31.5	87	31.2	657	26.8
60–69	72	9.9	68	12.4	140	15.6	32	11.5	312	12.8
> 70	29	4.0	25	4.6	36	4.0	23	8.2	113	4.5
Family History of Diabetes										
Yes	265	36.5	199	36.4	402	44.6	107	38.4	994	40.5
No	461	63.5	348	63.6	499	55.4	172	61.6	1459	59.5
Current or Previous Smoking History										
Yes	88	12.1	55	10.1	32	3.6	37	13.3	212	8.6
No	638	87.9	492	89.9	869	96.4	242	86.7	2241	91.4
Ethnicity										
Sindhi	29	4.0	9	1.6	15	1.7	9	3.2	62	2.5
Balochi	09	1.2	5	0.9	8	0.9	4	1.4	26	1.1
Punjabi	21	2.9	18	3.3	25	2.8	20	7.2	84	3.4
Pathan	127	17.5	89	16.3	96	10.7	111	39.8	423	17.2
Muhajir	518	71.3	367	67.1	734	81.5	110	39.4	1729	70.5
Other	22	3.0	59	10.8	23	2.6	25	9.0	129	5.3
History of Hypertension										
Yes	210	28.9	75	13.7	273	30.3	171	61.3	728	29.7
No	516	71.1	472	86.3	628	69.7	109	38.7	1725	70.3

### Data management and analysis

Data on DM and TB verbal screening results, referrals, and DM test results was recorded using a custom-built mobile-phone application. Other variables ascertained at baseline included family-history of diabetes, smoking history, ethnicity, and past history of TB treatment (Table 2). Patients’ chest X-ray and Xpert MTB/RIF results at the diagnostic centers were recorded using OpenMRS, an Open source enterprise electronic medical record system platform. The mobile and web applications included several data validation checks to ensure data-accuracy. Field supervisors and project management staff were responsible for overall data-validation and accuracy including reporting to the NTP.

**Table 2:** Baseline characteristics and results of glucometer testing conducted on TB patients through community-based TB-DM screening

	Persons with TB Tested for DM*		Pre-Diabetes*		Newly Diagnosed DM cases		*p*-value†
	*n*	%	*n*	%	*n*	%	
Total	266	100	25	100	8	100	
Sex							
Male	108	40.6	13	48.0	4	50.0	0.99
Female	158	59.4	12	48.0	4	50.0	
Age (years)							
< 20	60	22.6	4	16.0	0	0.0	< 0.01
20–29	93	35.0	5	20.0	0	0.0	
30–39	43	16.2	2	8.0	1	12.5	
40 and above	67	25.2	14	56.0	7	87.5	
Family History of DM*							
Yes	32	12.0	1	4.0	3	37.5	0.97
No	234	88.0	24	96.0	5	62.5	
Current or Previous Smoking History							
Yes	21	7.9	7	28.0	2	25.0	< 0.01
No	245	92.1	18	72.0	6	75.0	
Ethnicity							
Sindhi	14	5.3	2	8.0	2	25.0	0.11
Balochi	2	0.8	0	0.0	0	0.0	
Punjabi	8	3.0	1	4.0	0	0.0	
Pathan	118	44.4	5	20.0	3	37.5	
Muhajir	91	34.2	12	48.0	1	12.5	
Other	33	12.4	3	12.0	1	12.5	
MTB Burden							
Negative (clinically diagnosed)	28	10.5	3	12.0	2	25.0	0.85
Very low-low	90	33.8	9	34.0	2	25.0	
Medium	90	33.8	9	36.0	3	37.5	
High	58	21.8	4	16.0	1	12.5	
Type of TB							
New	229	86.1	22	88.0	7	87.5	0.75
Retreatment	37	13.9	3	12.0	1	12.5	

^*^The cut-offs for RBS testing were taken from the International Diabetes federation

† The p-value denotes the level of significance found when testing differences in sociodemographic characteristics between individuals identified as new DM cases and those identified as pre-DM, among all TB patients who had a glucometer blood sugar test

Frequency analysis of baseline demographic characteristics of patients diagnosed with DM and Pre-DM/Impaired Fasting and TB were performed. The proportions of individuals diagnosed with Pre-DM and DM that had TB symptoms and subsequently underwent Xpert MTB/RIF testing and diagnosed with TB were calculated. The proportion of newly diagnosed DM cases among TB patients was calculated. Differences between the DM and Pre-DM groups were assessed for statistical significance using chi-square or Pearson’s test (Table 2). All statistical analysis was carried out using Stata 12.1 (College Station, TX: StataCorp LP).

The primary study outcomes were uptake of TB testing and uptake of DM testing. Overall uptake of TB testing, overall uptake of DM testing, uptake of TB testing and uptake of DM testing among patients presumptive for both DM & TB were computed (Fig. 1). Additionally, uptake of TB testing among pre-diabetic and diabetic patients was assessed by comparing the proportion of pre-diabetic and diabetic individuals that underwent Chest X-ray and Xpert MTB/RIF testing to the proportion within each arm who were presumptive for TB. Uptake of DM testing among MTB positive patients was computed by comparing the proportion of MTB positive patients who underwent a glucometer-based random blood sugar test to the proportion within that arm who were presumptive for DM. The yield of TB among individuals identified with Pre-DM and DM was computed as the proportion of individuals who tested positive for TB among all individuals with DM and pre-DM who underwent TB testing via chest X-ray and Xpert MTB/RIF in this project. The yield of DM among individuals diagnosed with TB was calculated as the proportion of individuals identified with DM and Pre-DM among all individuals with TB who underwent glucometer-based RBS testing in this project.

## Results

A total of 450,385 individuals were verbally screened during the study period. Of those verbally screened, 3559 (0.8%) were identified as having presumptive DM, 14,550 (3.2%) with both presumptive DM and TB, and 75,587 (16.8%) as having presumptive TB only (Fig. 1). The overall uptake of DM testing among those identified with presumptive diabetes was 26.1% while the overall uptake of TB testing among those with presumptive TB was 5.9% (Fig. 1). Individuals with a presumptive diagnosis of both DM and TB were referred for both a glucometer based random blood sugar test and a Chest X-ray and Xpert MTB/RIF test. Among the group that was presumptive for both DM and TB, the uptake of DM testing was 12.5% and the uptake of TB testing was 24.2% (Fig. 1).

### Baseline socio-demographic and clinical characteristics of pre-diabetic and diabetic patients

The majority of pre-diabetic and diabetic patients self-identified as Muhajir (the most common ethnic group in Karachi). Of those patients who tested positive for diabetes, 547 (31.6%) were newly diagnosed. Of the 1180 previously known diabetes cases, 901 (76.4%) had blood sugar levels above the recommended range for optimal diabetics control (Table 1). A higher proportion of females were identified with pre-diabetes and diabetes (58.1%) and a family history of diabetes, history of smoking and history of hypertension was reported in 40.5, 8.6 and 29.7% of diabetics respectively. A history of hypertension was reported in a higher proportion of previously known diabetics with controlled blood sugar levels (61.3%) compared to known diabetics with poorly controlled blood sugar levels (30.3%), newly diagnosed diabetics (13.7%) and pre-diabetics (28.9%) (Table 1).

### Baseline socio-demographic characteristics of TB patients

The yield of diabetes among TB patients was found to be significantly higher in patients > 40 years of age (*p*-value < 0.01) and among smokers compared to non-smokers (p-value < 0.01) (Table 2). Gender, MTB burden, ethnicity and past history of TB treatment were not associated with an increased yield of DM (Table 2).

### Uptake of diabetes testing

A total of 4722 (26.1%) individuals with presumptive DM underwent glucometer testing, with 726 (15.4%) identified as pre-diabetic (198 of whom had both presumptive TB and DM), and 1727 (36.6%) diagnosed with diabetes (432 of whom had both presumptive DM and TB). Among those diagnosed with TB, a higher proportion of those with presumptive DM consented for DM screening using a glucometer, than those without presumptive DM (33.8% vs 14.7%, *p* < 0.001). Among those who underwent glucometer testing, 25 (9.4%) were identified as pre-diabetics and 8 (3%) as diabetics. Of those diagnosed with diabetes and pre-diabetes, 100 and 56% (14/25) had presumptively screened positive for DM (Fig. 1). The median age of individuals identified as presumptive for TB and DM was 34 years (IQR 25–45), and 47 years (IQR 38–55) respectively. The proportion of females among those identified as presumptive for TB and DM, was 47.4 and 50.1% respectively.

### Uptake of TB testing

A high proportion (22.8%) of individuals with presumptive TB, tested positive for MTB on Xpert MTB/RIF. Overall, the uptake of screening for tuberculosis was quite low among prediabetics and diabetics. 6.6% of prediabetic cases and 3.9% of diabetic cases underwent TB testing via Chest X-ray and Xpert MTB/RIF. However, the uptake was significantly higher among those who had presumptive TB (22.2% vs 0.8% among pre-diabetics; and 13.7% vs 0.62% among diabetics). Of the 153 who consented for TB testing, 153 had a chest X-ray and among these 115 (75.2%) underwent Xpert MTB/RIF testing (the remainder were unable to expectorate sputum and excluded from further diagnostic evaluation) (Table 3). A total of 8 pre-diabetics (16.7%) and 19 diabetics (28.4%) tested positive for TB on Xpert MTB/RIF testing (Fig. 1).

**Table 3:** Xpert MTB/RIF test results of individuals diagnosed with Diabetes and Pre-Diabetes through community-based screening

	Individuals identified with Pre-Diabetes		Individuals identified with Diabetes Mellitus		Total	
	Number(*n*)	Percent (%)	Number (*n*)	Percent (%)	Number (*n*)	Percent (%)
Total	726	100	1727	100	2453	100
Underwent Paid Chest X-Ray	62	8.5	91	5.3	153	6.2
Underwent Xpert MTB/RIF	48	77.4	67	73.6	115	75.2
Diagnosed with TB	8	16.7	19	28.4	27	23.5
Started on treatment for TB	8	100	19	100	27	100

## Discussion

Our study evaluated bi-directional TB-DM screening in a high TB and DM prevalence setting. In this study, the yield of TB among those with diabetes and pre-diabetes was 23.5% while the yield of pre-DM/DM among individuals who had tested positive for TB was 12.4% (Fig. 1). However, it is important to note that the yield of TB in our sample has a great risk of selection bias. Notably all had presumptive TB; the number of diabetics without presumptive TB who took up screening was too small to comment on the yield of TB among those tested for TB without any suggestive symptoms. A number of studies, as well as the Pakistan TB prevalence survey have identified a large proportion of TB cases with only Chest X-ray abnormalities and no TB symptoms supporting its use as the front-line screening procedure [15]. Therefore, we aimed to test all individuals for TB using chest radiography, regardless of symptoms. In circumstances where chest X-rays are unavailable symptomatic screening can be used as an alternative. In this study, a high yield of TB was recorded among those with TB symptoms. We were unable to assess the effectiveness of the chest X-ray in identifying additional cases of TB as the majority of cases referred were also symptomatic for TB.

While our study supports the growing body of literature highlighting the association between TB and DM [8] it primarily identifies a number of operational constraints that programs may need to address during larger scale TB-DM screening interventions. A high yield of TB was found among the diabetics and pre-diabetics within our study population. The uptake of diagnostic evaluation for TB was low, and mainly confined to those who had symptoms suggestive of TB. In addition, since physician prescriptive practices also significantly affect patient behavior, it is possible that only diabetics with clinical suspicion of TB were actively referred [16]. Additional constraints in testing diabetics for TB included the price for the chest X-ray and the distance to the centers for diagnostic evaluation. Our experience suggests that in a pay for testing model only diabetics with a high suspicion of TB will be referred, as both providers and patients perceived the costs and time associated with the X-ray as key barriers. Also, approximately a quarter of patients who agreed to undergo a chest X-ray, could not be tested on Xpert MTB/RIF due to their inability to expectorate sputum and this further limited the number of diabetics among whom the yield of TB could be assessed.

Our early experience of implementing TB-DM screening in a programmatic setting in the private-sector offers a number of lessons for potential interventions that can be carried out at scale. Firstly, physicians must be trained to refer diabetics for routine screening for TB, as is carried out for other diabetes-associated complications such as nephropathy or retinopathy. Patients also need to be appropriately counseled regarding the risks of developing TB, particularly those with poorly controlled diabetes since they are at higher risk for developing TB [17]. Secondly, user-fees for the X-ray and distance to the TB centers limited the number of diabetics undergoing TB screening in our program. It can be assumed that user fee is likely a barrier to uptake of preventive health services such as screening for TB and DM. As a preventative intervention with potential public health benefits, cost-free or subsidized services may increase uptake of TB testing. This may likely encourage pre-diabetics and diabetics to undergo screening, particularly those who have no symptoms of TB, and will help decrease the case-detection gap for TB. Public health practitioners and program managers that are eager to roll out a bi-directional TB-DM screening program in the private sector, should incorporate greater cost subsidies for patients in the program budget. Third, scale-up of TB screening in diabetics will require ensuring adequate sputum expectoration from diabetics through use of nebulizers and appropriate counseling. Commercial labs in the private-sector and most healthcare providers do not routinely carry out such procedures and will require capacity-strengthening. Existing specialized diabetes treatment centers with high volumes of known diabetics or private-practices of diabetes and endocrine specialists can be targeted to refer diabetics for TB screening.

Over 50% of diabetics and pre-diabetics identified in our program were newly diagnosed further highlighting the burden of undiagnosed diabetes and the need to introduce affordable and accessible diabetes screening in the private-sector at scale as a prelude for enhanced case-detection for TB.

Screening for DM among TB patients presented fewer operational challenges in our model as patients were offered screening during the time of registration for TB treatment. The yield of pre-DM and DM identified among individuals with TB in our program was higher (12.4%) than the DM prevalence in the general population of Pakistan (6.9%) [8]. This is consistent with findings from similar studies from India that have reported a prevalence between 5 and 54.1% of DM in TB patients [18–20]. Routine screening for DM among TB patients should therefore be considered at TB management facilities in Pakistan.

Diabetes screening for TB patients can be best carried out at the time of initiation of TB treatment and registration. Low-cost, point-of-care tests for diabetes are recommended as patients are more likely to consent to them although their results are less accurate. Interventions will need to ensure that these tests are available either at laboratories or at provider clinics where TB treatment is initiated for private-sector patients and data on diabetes screening and control is captured as part of the TB surveillance system. Integrated delivery units such as the ones developed by our project offer a potential solution where TB diagnostics, DM screening and TB-DM treatment can be offered in one facility with supporting systems for data capture and monitoring of outcomes.

## Conclusion

This study provides evidence for the operationalization and the associated challenges of bi-directional screening of TB and DM in the private health sector of low-middle income countries. A high yield for TB was identified among diabetics and pre-diabetics in our study population, however the overall uptake of bi-directional screening remained low. A large number of diabetics were newly identified suggesting the need for implementation of collateral active case-finding initiatives for DM. This study also provides a potential model for integration of TB diagnostics, DM screening and TB-DM treatment within one facility that can be similarly adapted for other areas of public health. The private sector can be engaged cost-effectively through trainings and incentives to help achieve increased case-detection for both TB and DM.

## Abbreviations

DM: Diabetes Mellitus
FBS: Fasting Blood Sugar
IDF: International Diabetes Federation
MTB: *Mycobacterium tuberculosis*
NCD: Non-Communicable Disease
NNT: Numbers needed to test
NTP: National Tuberculosis Program
Pre-DM: Pre-Diabetes Mellitus
PTP: Provincial Tuberculosis Program
RBS: Random Blood Sugar
TB: Tuberculosis
WHO: World health Organization
